# A small, polyphyletic group of *Firmicutes* synthesizes trimethylamine from l‐carnitine

**DOI:** 10.1002/mlf2.12079

**Published:** 2023-09-13

**Authors:** Marius Vital, Ylenia Heinrich‐Sanchez

**Affiliations:** ^1^ Hannover Medical School Institute for Medical Microbiology and Hospital Epidemiology Hannover Germany

## Abstract

Gut microbiota‐derived trimethylamine (TMA) is associated with cardiometabolic disorders and exemplifies a microbial involvement in the etiology of emerging, noncommunicable diseases, the leading causes of death worldwide. Three biochemical pathways taking dietary compounds as intake have been described with distinct taxa involved that are all present at low relative abundances. A recently discovered pathway is now considered to be the main route for TMA synthesis from l‐carnitine involving γ‐butyrobetaine as an intermediate product. By comprehensive (meta) genomic screening of publicly available data, namely, genomes of the UHGG catalog (*n* > 200,000) and 10 metagenomic (transcriptomic) data sets, we revealed bacteria synthesizing TMA via this pathway and specified their ecophysiology. Results will contribute to stratification of individuals based on their gut microbiota's potential to synthesize TMA and might aid in the development of strategies restricting TMA formation.

Trimethylamine (TMA) is formed by gut bacteria and subsequently oxidized by hepatic flavin‐containing monooxygenase 3 into trimethylamine N‐oxide (TMAO), which is associated with cardiometabolic and renal disorders, independent of traditional risk factors and in a dose‐dependent manner^1^, ^2^, ^3^, ^4^ (Figure 1A). TMAO acts via various pathomechanisms, such as the formation of foam cells^5^, enhancement of platelet hyperreactivity^6^ and activation of proinflammatory cascades^7^. TMA is synthesized from diverse dietary precursors, mainly betaine, l‐carnitine and choline, via distinct biochemical routes involving a multitude of different taxa^8^. For l‐carnitine, which is primarily enriched in red meat^9^, ^10^, a microbial‐encoded pathway catalyzing the reaction consisting of a two‐component Rieske‐type oxygenase/reductase (cntAB) was discovered in 2014^11^, which is primarily encoded on *Enterobacteriaceae* of human gut microbiota^12^. However, due to its oxygen requirement and lack of transcription of those genes in the colon, this pathway is now not considered to contribute to the TMA pool in anoxic gut environments^8^. In 2018, Koeth and colleagues indicated that l‐carnitine can be anaerobically converted into TMA via an alternative two‐step cascade with the intermediate γ‐butyrobetaine (γBB)^13^. While the initial step, that is, the formation of γBB, is performed by multiple taxa showing the *cai* operon, the subsequent conversion into TMA is catalyzed by enzymes encoded on the γBB utilization (*bbu* or *gbu*) gene cluster that has been recently discovered^14^, ^15^. For investigating the latter step, *Emergencia timonensis* served as the model organism, and heterologous expression in *Escherichia coli* of four of the six genes, namely, *bbuA, B, C*, and *E*, was shown to be sufficient for the formation of TMA from γBB^15^. Screening of genomes for exhibiting *bbu* genes has revealed a few additional candidates, such as *Agathobaculum desmolans* that was isolated from cat feces^14^, ^15^. Additionally, *bbuA* homologs were shown to be widely distributed in metagenomes of gut microbiota of publicly available data sets^14^ and specifically enriched in subjects consuming red meat during a dietary intervention study^15^.

**Figure 1:**
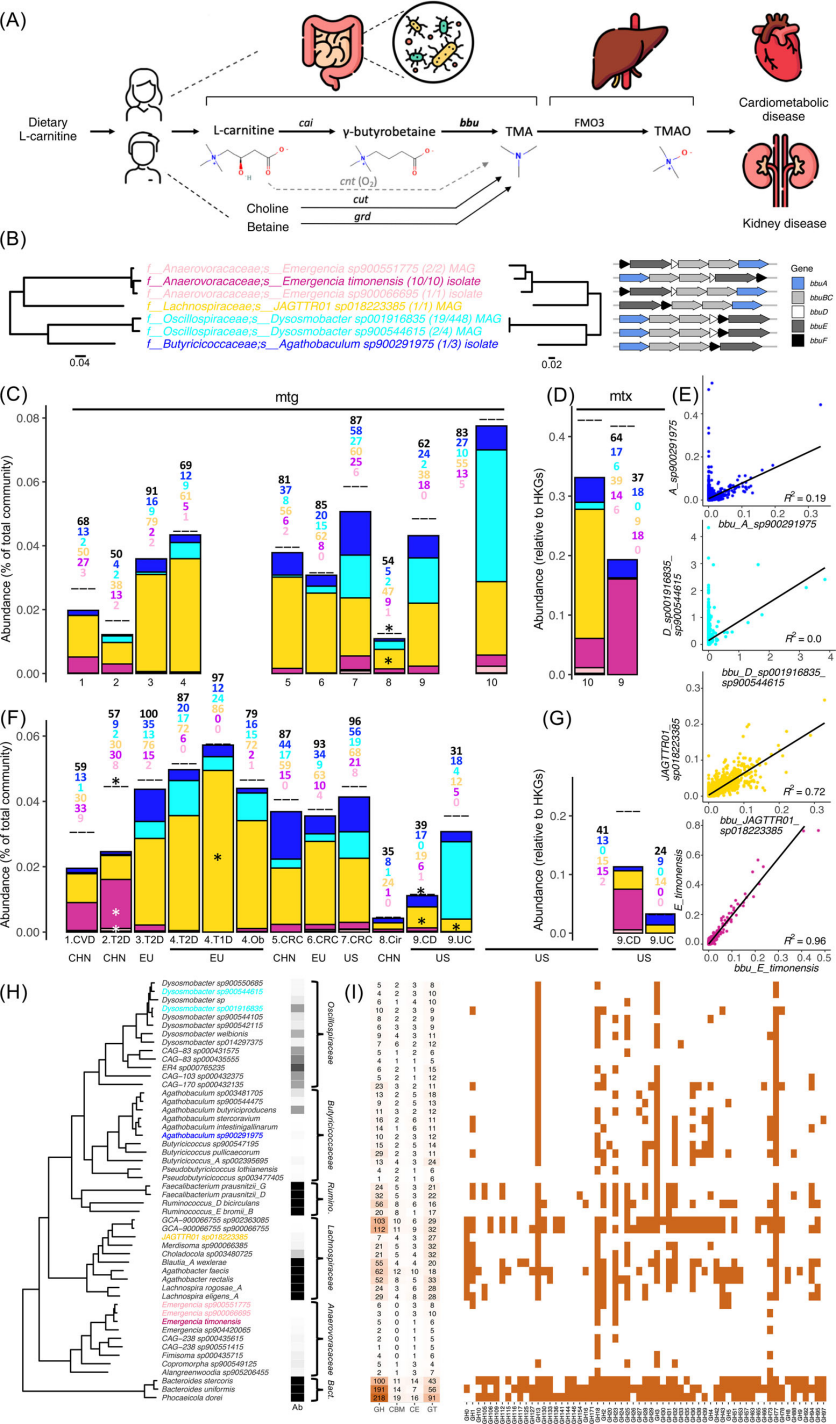
Characterization of bacteria that contain the γ‐butyrobetaine utilization (*bbu*) gene cluster for the formation of trimethylamine (TMA). (A) An overview of TMA(O) generation and its association with disease. (B) A phylogenetic tree (based on housekeeping genes [HKGs]) of bacteria showing the *bbu* gene cluster from the UHGG catalog, along with their taxonomic affiliations. Numbers in brackets show the amount of genomes carrying the pathway and the total genomes of the species, respectively, and its nature, specified as metagenome‐assembled genome (MAG) or isolate, is given as well. The second tree in the middle of the panel is based on concatenated *bbu* gene sequences, and their arrangements are shown on the right. (C and D) Average overall abundances of *bbu* genes (black dash lines) and of pathways associated with individual species in healthy control subjects of 10 different metagenomic (mtg) (C) and two metatranscriptomic (mtx) (D) data sets; for detailed information on data sets, see Table S1. Numbers indicate the prevalence, that is, the percentage of samples, where total *bbu* genes (black) and those associated with individual pathway‐carrying taxa were detected. (E) Correlations of *bbu* pathway abundances with individual taxa abundances (based on HKGs) in those samples. (F and G) Average relative abundances and expression levels, respectively, of *bbu* genes and associated taxa carrying the pathway in subjects suffering from various diseases. Statistical analyses were performed (an asterisk marks significant (*p* < 0.05) abundance differences based on logistic regression analysis). (H) Carbohydrate‐active enzymes (CAZyme) repertoires of *bbu* pathway carriers along with related taxa from the same taxonomic families. Representative genomes of additional abundant species of the *Bacteroidaceae* and *Ruminococcaceae* were included in the analyses for comparison. (I) Enzyme family spectra (presence/absence) for glycoside hydrolases (GHs). Ab, relative abundance; Bact., *Bacteroidaceae*; CBM, carbohydrate‐binding module; CD, Crohn's disease; CE, carbohydrate esterases; CHN, China; Cir, cirrhosis; CRC, colorectal cancer; CVD, cardiovascular disease; EU, Europe; FMO3, flavin‐containing monooxygenase 3; GT, glycosyltransferases; Ob, obesity; Rumino., *Ruminococcaceae*; T1/2D, type 1/2 diabetes; UC, ulcerative colitis; US, the United States.

Detailed investigations on bacteria converting γBB into TMA and their distribution in human gut microbiota are lacking. To this end, we performed comprehensive pathway screenings on the Unified Human Gastrointestinal Genome catalog (UHGG.v2.0) that comprises over 200,000 nonredundant genomes derived from isolates and metagenome‐assembled genomes (MAGs) of the gut environment^16^. We revealed 36 candidates that clustered into seven different species associated with five families of the *Firmicutes* (Figure 1B). Genomes of *E. timonensis*, *E. sp900066695*, and *Agathobacter sp900291975* were derived from isolates, whereas *bbu* genes associated with other candidates were found on MAGs. Most candidates showed all six genes; *JAGTTR01 sp018223385 (JAGTTR01)* and *A. sp900291975* were lacking *bbuD*, which is, however, not considered essential for the reaction^15^. Clustering of concatenated *bbu* genes reflected phylogenetic relatedness based on housekeeping genes (HKGs) (Figure 1B), which was also true when investigating *bbu* genes individually (Figure S1). These results suggest that genes have been transmitted laterally and not via horizontal gene transfer between taxa.

We screened 10 publicly available metagenomic data sets of subjects from three different regions—China, Europe, and North America—for abundances of the *bbu* gene cluster and associated taxonomies (Figure 1C–G). Data sets involved subjects diagnosed with cardiovascular disease (CVD), type 1 and type 2 diabetes (T1D/T2D), obesity (Ob), colorectal cancer (CRC), cirrhosis (Cir), ulcerative colitis (UC), Crohn's disease (CD) and respective healthy controls; two metatranscriptomic data sets were included as well (Table S1). In healthy controls, average relative abundances of the *bbu* genes were well below 0.1% of the total gut microbiota in all data sets (Figure 1C). It should be noted that low relative abundances of features of gut microbiota do not imply irrelevance. Many functions that play central roles for host health, such as the generation of secondary bile acids, the formation of hydrogen sulfide, and the other TMA‐synthesis pathways that act on choline and betaine, are all encoded on a small fraction of gut microbiota members^8^. Furthermore, in absolute terms, a relative abundance of 0.1% refers to 10^8^–10^9^ bacteria per gram stool, which can be considered fairly concentrated. Most of the *bbu* gene relative abundances were explainable by individual taxonomies, with *bbu* gene clusters from *JAGTTR01* comprising the majority. Pathways associated with *A. sp900291975, Dysosmobacter* species, and *E. timonensis* were also detected, while those linked to other *Emergencia* species played a neglectable role in all data sets (Figure 1C). Metatranscriptomic data indicated low expression of *bbu* genes, relative to all HKGs, and those linked to *JAGTTR01* (data set 10) and *E. timonensis* (data set 9) were dominating (Figure 1D). The prevalence, that is, the percentage of samples positive for *bbu* genes, ranged from 50% (data set 2) to 91% (data set 3). Sequencing depths between data sets were in a similar range for all studies (Table S1), and lower percentages of *bbu*‐positive samples in certain studies, especially in studies 2 and 8, cannot be attributed to sequencing depth. However, within studies, the absence of *bbu* genes did correlate with lower sequencing depth in five data sets (Figure S2), suggesting that the sequencing depth provided does not allow to prove the true absence of *bbu* genes, a common problem for low abundant features in bacterial communities. While relative abundances might, hence, be more homogeneous between samples if all were sequenced at high and equal depths, we think that the main trends on relative abundances and associated taxonomies of the *bbu*‐carrying community are reflected in the results of this study. The *JAGTTR01‐*associated gene cluster was the most prevalent (found in 38%–79% of all samples), whereas those associated with other taxa were found less frequently (Figure 1C). Correlation analyses between *bbu* gene relative abundances and respective taxa abundances (based on HKGs) showed strong associations for *JAGTTR01* (*R* ^2^ = 0.72) and *E. timonensis* (*R* ^2^ = 0.96) (Figure 1E), reflecting the fact that all genomes of these taxa showed the pathway (Figure 1B). For *A. sp900291975*, a much weaker correlation (*R* ^2^ = 0.19) was found, and relative abundances of *bbu* genes linked to the two *Dysosmobacter* species were not associated with their overall relative abundances as they often comprised >1% of the total community (Figure 1E). Results agree with the discordant distribution of *bbu* genes in these species (Figure 1B) and strengthen function‐centric analyses over plain compositional investigations when assessing gut microbiota's potential to form TMA from l‐carnitine. Given that the main player, namely, *JAGTTR01*, was a MAG and the only representative of its genus, we took a closer look at this genome to exclude technical issues leading to misassembly/wrong binning. Relative abundances of all its individual HKGs were strongly correlating with each other, and with *bbu* genes, indicating that the constructed genome is real and carries the pathway (Figure S3). However, if the bacterium indeed converts γBB into TMA still needs to be biochemically verified, which applies also for other MAGs revealed in this study. The retrieval of only one genome for *JAGTTR01* is most probably due to the fact that this taxon was consistently low abundant (only one sample showed the bacterium at a relative abundance >0.3% of the total community), providing too little coverage for assembly and binning. This was also true for *A. sp900291975*, where only three genomes were available (the one carrying the *bbu* cluster derived from an isolate), whereas several samples comprised *Dysosmobacter* species at higher relative abundances (>1% of the total community, Figure 1E), facilitating the construction of MAGs.

*Bbu* gene relative abundances in diseased individuals were similar to healthy controls in most data sets (Figure 1F). In data set 2, subjects suffering from T2D displayed higher relative abundances that were driven by *Emergencia* spp. associated pathways. T2D is indeed linked to higher TMAO plasma concentrations^17^. However, TMA‐generating pathways from betaine and choline are also increased in subjects suffering from T2D^8^, and the contribution of the *bbu* pathway to the total TMA pool still remains to be elucidated. Given its specific association with red meat intake, it seems likely that these bacteria play an important role in TMA generation in individuals fostering respective diets^9^, ^10^, and its contribution to the total TMA pool and disease development thus needs to be assessed in a diet‐dependent manner. *Bbu* genes were less abundant in samples from cirrhotic patients and those suffering from CD, with *JAGTTR01* showing lower concentrations, whereas *bbu* genes linked to the taxon were increased in T1D. For cases of CD and UC, lower expression levels of *bbu* genes were found that did, however, not reach significant thresholds (Figure 1F).

Little is known about the ecophysiology of revealed key players and we aimed to characterize their substrate spectra in more detail by applying carbohydrate‐active enzymes (CAZyme) analyses^18^. In the gut, bacteria do usually not grow on a single carbon source but feed on a multitude of substrates. This is exemplified by bacteria that form TMA from choline, which contain a multitude of CAZymes and whose abundances were associated with diets containing polysaccharide‐rich foods^12^, ^19^. Bacteria under study feed on l‐carnitine, which does not, however, automatically imply that meat‐derived substances are solely defining their substrate spectra. Insights into the capacity to degrade complex polysaccharides, the main carbon/energy source in the colon, are thus crucial for understanding their ecophysiology. For comparison, we included related taxa from the same families as well as selected abundant key members of gut microbiota. In agreement with their low relative abundances, all *bbu* pathway carriers showed only a few CAZymes, especially the category glycoside hydrolases (GHs), which are key for degradation of complex carbohydrates (Figure 1H). While major gut members, such as *Agathobacter* spp., *Bacteroides* spp., and *Faecalibacterium* spp., displayed dozens to >100 genes assigned as GHs, none of the genomes under study displayed >10 enzymes from this category, similar to carbohydrate‐binding modules (CBMs) that play important roles in extracellular polysaccharide degradation^20^. Very few CBMs were found on *bbu* carriers, and *Emergencia* completely lacked these genes. With a few exceptions, the low capacity for degrading complex carbohydrates was conserved within *Oscillospiraceae*, *Butyricicoccaceae*, and *Anaerovoracaceae*, which was coherent with low abundances of most members of these families. In *Lachnospiraceae*, which includes *JAGTTR0*, several highly abundant members, such as *Agathobacter* and *Blautia_A*, displaying a multitude of GHs were detected. Two species that were most closely related to *JAGTTR0* and that consisted of only one MAG showed >100 GHs, despite representing very low abundant members of gut microbiota (Figure 1H). Along with the low amounts of the total GHs for *bbu*‐displaying bacteria, their spectra of GH families were limited as well (Figure 1I). GH18, which is involved in chitin degradation, was found on all *bbu*‐displaying taxa, except for *D. sp900544615*. The widely distributed GH13 and the related GH77, which are both acting on α‐glucoside linkages and are involved in starch degradation, were found in all *bbu* carriers, except for *Emergencia* species. *A*. sp900291975 and *Dysosmobacter* species also carried GH3, a widely distributed exo‐acting enzyme with broad substrate specificities. The overall low abundance of GHs is in contrast to TMA‐producing bacteria acting on choline that show a multitude of GHs^12^.

In summary, our analyses suggest that revealed bacteria are ubiquitously present, however, at low relative abundances and with a limited potential to degrade complex carbohydrates. These characteristics indicate that these bacteria have narrow nutritional niches and the conversion of γBB into TMA might, hence, be an important part of energy generation. Our study enlarged the target spectrum of taxa forming TMA, aiding risk assessment, where individuals are stratified based on their potential to synthesize this toxic compound. This might further contribute to the development of precision strategies based on dietary interventions to restrict TMA formation from l‐carnitine.

## AUTHOR CONTRIBUTIONS

**Marius Vital**: Conceptualization (lead); formal analysis (lead); funding acquisition (lead); investigation (lead); methodology (lead); project administration (lead); visualization (lead); writing—original draft (lead); writing—review and editing (lead). **Ylenia Heinrich‐Sanchez**: Conceptualization (supporting); methodology (supporting); validation (supporting); visualization (supporting); writing—original draft (supporting); writing—review and editing (supporting).

## ETHICS STATEMENT

This study did not involve any human participants or animal subjects.

## CONFLICT OF INTERESTS

The authors declare no conflict of interests.

## Supporting information

Supporting information.

Supporting information.

Supporting information.

Supporting information.

## Data Availability

All data are publicly available (see Table S1 for references of individual metagenomic data sets).
