# Supplementary material for: A small, polyphyletic group of Firmicutes synthesizes trimethylamine from l‐carnitine
Source: mLife. 2023 Sep 13;2(3):267–71. doi: 10.1002/mlf2.12079 (PMC10989800; doi:10.1002/mlf2.12079)
Supplement: Supplementary file 1 — Supporting information. [file MLF2-2-267-s004.docx]

**Supporting Information**

**Materials and Methods**

*Screening genomes for exhibiting the* bbu*-gene cluster*

Hidden Markov Models (HMM) were built based on alignments of *bbu* genes from reference (1). Due to sequence similarity between *bbuB* and *bbuC* one model was constructed comprising both genes. Subsequently, all genomes of the Unified Human Gastrointestinal Genome catalogue (UHGG.v2.0) (2) that displayed >80 % completeness and <5% contamination were screened (n=205,303), where obvious HMM score drops served as cut-offs. Candidates that exhibited all genes essential for the conversion of γ-butyrobetaine (γBB) to TMA, namely *bbuA,BC,E,* were included (n=36) in follow-up analyses. A phylogenetic tree was constructed on house-keeping genes (HKGs) via the Genome Taxonomy Database-toolkit (GTDB-Tk) pipeline (v2.1.0; *gtdbtk infer*), which also served for assigning taxonomies (release 207) (3). Additionally, a tree was built from concatenated and aligned (via Clustal Omega (www.ebi.ac.uk/Tools/msa/clustalo/)) *bbuA,B,C,E* genes using FastTree2 (v1.1.10) (4). Trees and gene arrangement were visualized in R using ggtree (v3.0.4) and gggenes (v0.4.1), respectively.

*Analysing metagenomic/transcriptomic datasets*

Raw reads from samples of datasets shown in Table S1 were downloaded from NIH’s Sequence Read Archive and filtered using fastp (v0.23.2; -5 20 -3 20 -l 70) (5). BBmap2 (*bbmap.sh*; https://jgi.doe.gov/data-and-tools/software-tools/bbtools/bb-tools-user-guide/bbmap-guide/) was used to map reads in paired-end mode to representative genomes of the UHGG.v2. To assure database comprehensiveness, one additional high-quality genome (randomly selected) for each species was included as well. In order to particularly check for *bbu* gene diversity, nucleotide sequences were clustered via CD-HIT (v4.7; *cd-hit-est*) at 95 % similarity and it was confirmed that each cluster was represented by at least two genomes if available. RPKM values were calculated (*pileup.sh* from BBmap2) and *bbu* gene abundances were subsequently recorded relative to the average abundance of all house-keeping genes, yielding *bbu* gene abundances as percentage of total genomes of a sample. Finally, average abundance of *bbu* genes was calculated and abundances of individual *bbu*-exhibiting taxa were determined, where all essential *bbu* genes had to be detected in order to consider a taxon as present. Data were visualized in R using ggplot2 (v3.3.5). Statistical analyses between diseased individuals and healthy controls were done based on logistic regression analysis using the *glm* function.

*Analyzing CAZyme repertoires*

dbCAN3 (v2.0.11; *run-dbcan*) was used to screen genomes (6), where Carbohydrate-Active enzymes (CAZymes) had to be detected by two of the three algorithms applied by the program for calling them present. Next to the revealed *bbu*-pathway carrying bacteria, representative genomes of all other species of the same genera were included into analyses; for *Dysosmobacter* all species displaying an average abundance of 0.01 % and for *JAGTTR01 sp018223385* the four most closest species (based on ANI values determined by FastANI (v1.33) (7)) were included. The additional five most abundant species associated with respective families as well as abundant taxa from distinct taxonomies were incorporated for comparison reasons. A tree was built from HKGs via GTDB-Tk and visualized, along with CAZyme repertoires, using ggtree. Interpretations are based on CAZypedia (available at http://www.cazypedia.org/).

*Chemical structures*

Gamma-BB : CID 725

https://pubchem.ncbi.nlm.nih.gov/compound/725#section=2D-Structure

L-carnitine: CID 10917

https://pubchem.ncbi.nlm.nih.gov/compound/10917#section=2D-Structure

TMA: CID 1146

https://pubchem.ncbi.nlm.nih.gov/compound/1146#section=2D-Structure

TMAO: CID 1145

https://pubchem.ncbi.nlm.nih.gov/compound/1145#section=2D-Structure

**Table S1.** Overview individual metagenomic/transcriptomic datasets included in the study. CVD: cardiovascular disease, T1(2)D: type 1(2) diabetes, CRC: colorectal cancer, mtg: metagenome, mtx: metatranscriptome, UC: ulcerative colitis, CD: Crohn´s disease, CHN: China, EU: Europe, US: United States of America. CHN/EU: fecal matter derived from European subjects, whereas sample processing was performed in China. Sequencing depth, i.e., amount of paired-end reads post-filtering (average ± stdev) are given as well.

| **Study** | **Reference** | | **Short description** | **Continent** | **Sequencing depth** |
| --- | --- | --- | --- | --- | --- |
| 1 | (8) | Jie *et al,* 2017 | CVD (n=159) vs. controls (n=117) | CHN | 2.55x10^7^ ± 5.76x10^6^ |
| 2 | (9) | Qin *et al*, 2012 | T2D (n=182) vs. controls (n=185) | CHN | 1.60x10^7^ ± 7.45x10^6^ |
| 3 | (10) | Karlsson *et al*, 2013 | T2D (n=53) vs. controls (n=43) | EU | 1.23x10^7^ ± 5.51x10^6^ |
| 4a | (11) | Le Chatelier *et al*, 2013 | Obese (n=153) vs. controls (n=106) | EU | 1.78x10^7^ ± 1.32x10^6^ |
| 4b | (12) | Forslund *et al*, 2015 | T2D (n=85), T1D (n=34) vs. samples from 4a | EU | 2.23x10^7^ ± 7.25x10^6^ |
| 5 | (13) | Feng *et al*, 2015 | CRC (n=46) vs. controls (n=63) | CHN /EU | 2.43x10^7^ ± 3.95x10^6^ |
| 6 | (14) | Zeller *et al*, 2014 | CRC (n=91) vs. controls (n=61) | EU | 2.08x10^7^ ± 1.00x10^6^ |
| 7 | (15) | Vogtmann *et al*, 2016 | CRC (n=52) vs. controls (n=52) | US | 2.64x10^7^ ± 7.28x10^6^ |
| 8 | (16) | Qin *et al*, 2014 | Cirrhosis (n=116) vs. controls (n=114) | CHN | 1.98x10^7^ ± 1.24x10^7^ |
| 9 | (17) | Schirmer *et al*, 2018 | mtg: UC (n=78), CD (n=145) vs. controls (n=55)  mtx: UC (n=21), CD (n=46) vs. controls (n=11) | US | 1.04x10^7^ ± 5.55x10^6^  8.97x10^6^ ± 7.39x10^6^ |
| 10 | (18) | Mehta *et al*, 2018 | mtg & mtx of 78 subjects (4 time points) | US | 7.77x10^6^ ± 3.07x10^6^  6.62x10^6^ ± 5.31x10^6^ |

**Figure S1.** Phylogenetic tree of all genes comprising the *bbu* gene cluster.

**Figure S2.** Amount of paired end reads (counts) per dataset stratified by absence (0) and presence (1) of *bbu* genes. *significantly different based on logistic regression.

**Figure S3.** Spearman correlations of house-keeping gene abundances of *JAGTTR01 sp018223385* with each other and with all *bbu* genes based on all metagenomic samples analysed.

**References**

1. Rajakovich LJ, Fu B, Bollenbach M, Balskus EP. Elucidation of an anaerobic pathway for metabolism of l-carnitine-derived γ-butyrobetaine to trimethylamine in human gut bacteria. Proc Natl Acad Sci U S A. 2021;118(32).

2. Almeida A, Nayfach S, Boland M, Strozzi F, Beracochea M, Shi ZJ, et al. A unified catalog of 204,938 reference genomes from the human gut microbiome. Nat Biotechnol. 2021;39(1):105–14.

3. Chaumeil P-A, Mussig AJ, Hugenholtz P, Parks DH. GTDB-Tk: a toolkit to classify genomes with the Genome Taxonomy Database. Bioinformatics. 2019;36(6):1925–1927.

4. Price MN, Dehal PS, Arkin AP. FastTree 2 - approximately maximum-likelihood trees for large alignments. PLoS One. 2010;5(3):e9490.

5. Chen S, Zhou Y, Chen Y, Gu J. Fastp: An ultra-fast all-in-one FASTQ preprocessor. Bioinformatics. 2018;34(17):i884–90.

6. Zhang H, Yohe T, Huang L, Entwistle S, Wu P, Yang Z, et al. DbCAN2: A meta server for automated carbohydrate-active enzyme annotation. Nucleic Acids Res. 2018;46(W1):W95–101.

7. Jain C, Rodriguez-R LM, Phillippy AM, Konstantinidis KT, Aluru S. High throughput ANI analysis of 90K prokaryotic genomes reveals clear species boundaries. Nat Commun. 2018;9(1):5114.

8. Jie Z, Xia H, Zhong S, Feng Q, Li S, Liang S, et al. The gut microbiome in atherosclerotic cardiovascular disease. Nat Commun. 2017;8:845.

9. Qin J, Li Y, Cai Z, Li S, Zhu J, Zhang F, et al. A metagenome-wide association study of gut microbiota in type 2 diabetes. Nature. 2012;490(7418):55–60.

10. Karlsson FH, Tremaroli V, Nookaew I, Bergström G, Behre CJ, Fagerberg B, et al. Gut metagenome in European women with normal, impaired and diabetic glucose control. Nature. 2013;498:99–103.

11. Le Chatelier E, Nielsen T, Qin J, Prifti E, Hildebrand F, Falony G, et al. Richness of human gut microbiome correlates with metabolic markers. Nature. 2013;500(7464):541–6.

12. Forslund K, Hildebrand F, Nielsen T, Falony G, Le Chatelier E, Sunagawa S, et al. Disentangling type 2 diabetes and metformin treatment signatures in the human gut microbiota. Nature. 2015;528(7581):262–6.

13. Feng Q, Liang S, Jia H, Stadlmayr A, Tang L, Lan Z, et al. Gut microbiome development along the colorectal adenoma–carcinoma sequence. Nat Commun. 2015;6:6528.

14. Zeller G, Tap J, Voigt AY, Sunagawa S, Kultima JR, Paul I, et al. Potential of fecal microbiota for early-stage detection of colorectal cancer. Mol Syst Biol. 2014;10:1–18.

15. Vogtmann E, Hua X, Zeller G, Sunagawa S, Voigt AY, Hercog R, et al. Colorectal cancer and the human gut microbiome: Reproducibility with whole-genome shotgun sequencing. PLoS One. 2016;11(5):1–13.

16. Qin N, Yang F, Li A, Prifti E, Chen Y, Shao L, et al. Alterations of the human gut microbiome in liver cirrhosis. Nature. 2014;513(7516):59–64.

17. Schirmer M, Franzosa EA, Lloyd-price J, Mciver LJ, Schwager R, Poon TW, et al. Dynamics of metatranscription in the inflammatory bowel disease gut microbiome. Nat Microbiol. 2018;3:337–46.

18. Mehta RS, Abu-ali GS, Drew DA, Lloyd-price J, Subramanian A, Lochhead P, et al. Stability of the human faecal microbiome in a cohort of adult men. Nat Microbiol. 2018;3:347–55.
