# Supplementary figures and images for: A small, polyphyletic group of Firmicutes synthesizes trimethylamine from l‐carnitine
Source: mLife. 2023 Sep 13;2(3):267–71. doi: 10.1002/mlf2.12079 (PMC10989800; doi:10.1002/mlf2.12079)

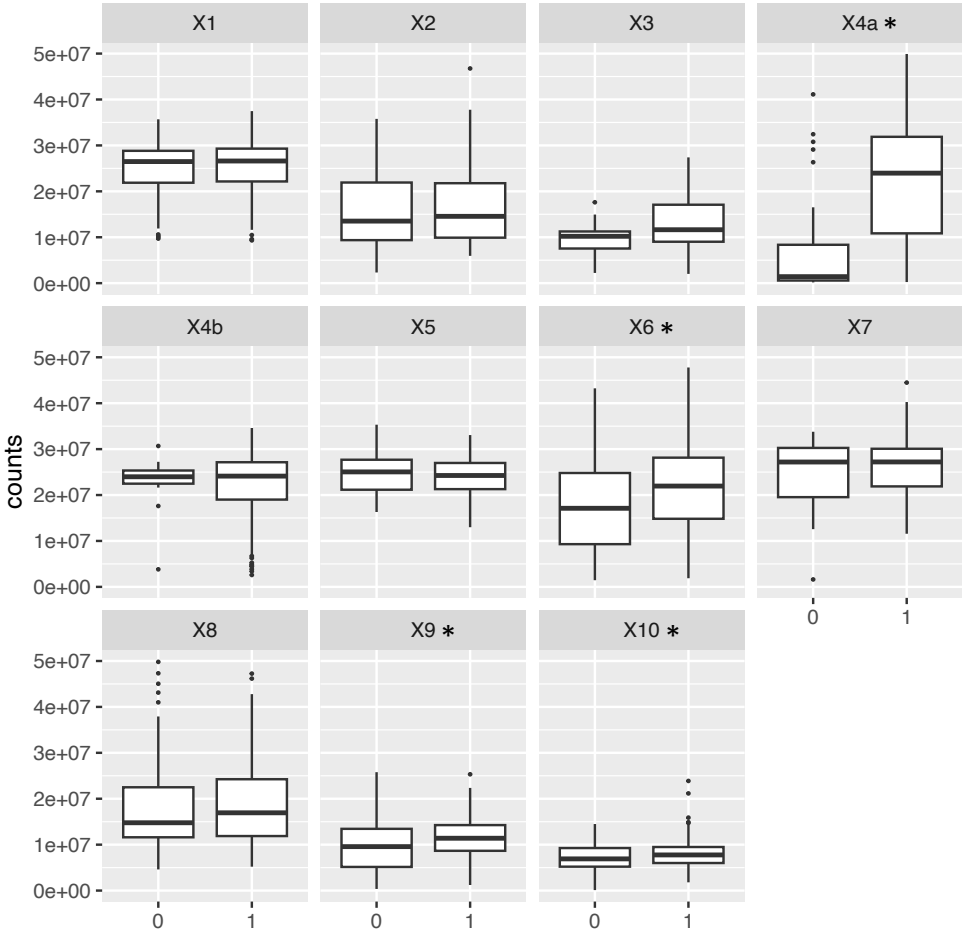

Supplement: Supplementary file 3 — Supporting information. [file MLF2-2-267-s002.pdf]

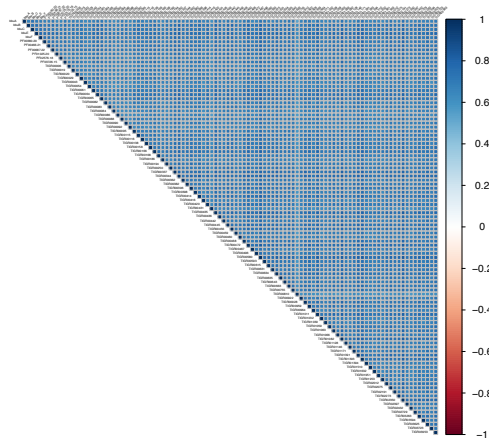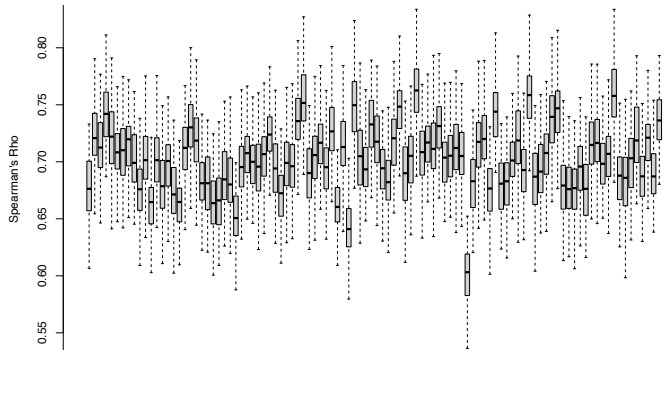

Supplement: Supplementary file 4 — Supporting information. [file MLF2-2-267-s003.pdf]
