# Supplementary material for: A small, polyphyletic group of Firmicutes synthesizes trimethylamine from l‐carnitine
Source: mLife. 2023 Sep 13;2(3):267–71. doi: 10.1002/mlf2.12079 (PMC10989800; doi:10.1002/mlf2.12079)
Supplement: Supplementary file 2 — Supporting information. [file MLF2-2-267-s001.pdf]

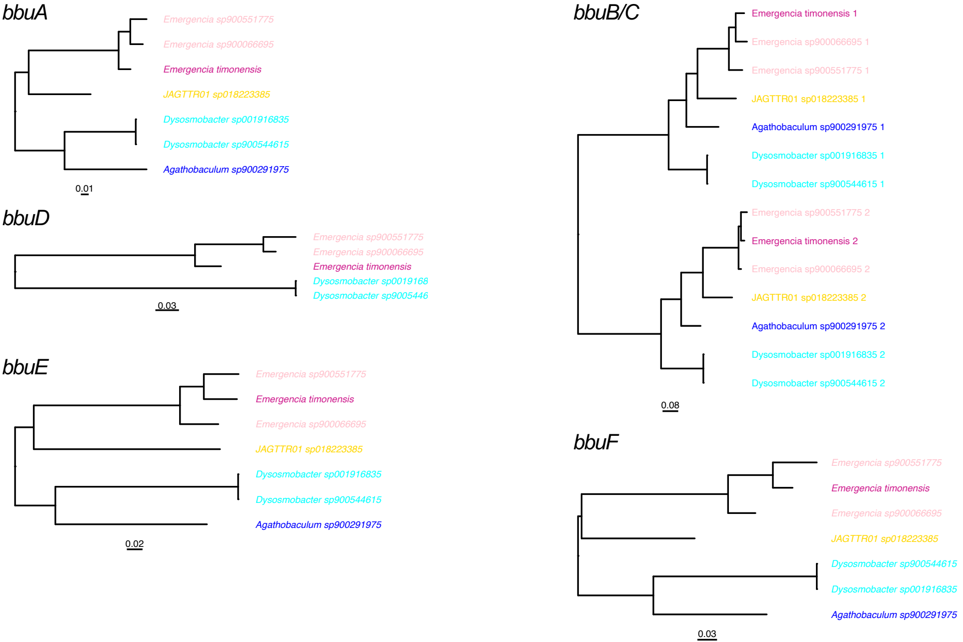

bbuA
Emergencia sp900551775
Emergencia sp900066695
Emergencia timonensis
JAGTTR01 sp018223385
Dysosmobacter sp001916835
Dysosmobacter sp900544615
Agathobaculum sp900291975
0.01
bbuD
Emergencia sp900551775
Emergencia sp900066695
Emergencia timonensis
Dysosmobacter sp0019168
Dysosmobacter sp9005446
0.03
bbuE
Emergencia sp900551775
Emergencia timonensis
Emergencia sp900066695
JAGTTR01 sp018223385
Dysosmobacter sp001916835
Dysosmobacter sp900544615
Agathobaculum sp900291975
0.02
bbuB/C
Emergencia timonensis 1
Emergencia sp900066695 1
Emergencia sp900551775 1
JAGTTR01 sp018223385 1
Agathobaculum sp900291975 1
Dysosmobacter sp001916835 1
Dysosmobacter sp900544615 1
Emergencia sp900551775 2
Emergencia timonensis 2
Emergencia sp900066695 2
JAGTTR01 sp018223385 2
Agathobaculum sp900291975 2
Dysosmobacter sp001916835 2
Dysosmobacter sp900544615 2
0.08
bbuF
Emergencia sp900551775
Emergencia timonensis
Emergencia sp900066695
JAGTTR01 sp018223385
Dysosmobacter sp900544615
Dysosmobacter sp001916835
Agathobaculum sp900291975
0.03
